# Discovery of three RNA viruses using ant transcriptomic datasets

**DOI:** 10.1007/s00705-018-4093-2

**Published:** 2018-11-10

**Authors:** Eleni Kleanthous, Ingrida Olendraite, Nina I. Lukhovitskaya, Andrew E. Firth

**Affiliations:** Division of Virology, Department of Pathology, University of Cambridge, Cambridge, UK

## Abstract

Three novel RNA viruses, named Formica fusca virus 1 (GenBank accession no. MH477287), Lasius neglectus virus 2 (MH477288) and Myrmica scabrinodis virus 2 (MH477289), were discovered in ants collected in Cambridge, UK. The proposed virus names were given based on the hosts in which they were identified. The genome sequences were obtained using *de novo* transcriptome assembly of high-throughput RNA sequencing reads and confirmed by Sanger sequencing. Phylogenetic analysis showed that Formica fusca virus 1 grouped within the family *Nyamiviridae*, Lasius neglectus virus 2 grouped within the family *Rhabdoviridae* and Myrmica scabrinodis virus 2 belongs to the family *Dicistroviridae*. All three viruses are highly divergent from previously sequenced viruses.

**Electronic supplementary material:**

The online version of this article (10.1007/s00705-018-4093-2) contains supplementary material, which is available to authorized users.

Insect-infecting viruses comprise a large and diverse group, but their full diversity has as yet been poorly investigated [1]. Further investigation may provide new insights into the long-term evolution and origin of viruses and may also reveal new tools for molecular biology research and biotechnology. Also, the host range of some of these viruses may make them suitable to use as biological control agents against invasive or pestiferous insect species.

We generated RNA-Seq libraries from ants sampled from various locations in Cambridge, UK [2]. Sequencing data have been deposited in ArrayExpress (http://www.ebi.ac.uk/arrayexpress) under the accession number E-MTAB-5781. Trimmed RNA-Seq reads were assembled *de novo* into contigs using TRINITY v2.3.2 [3, 4], and assembled contigs were compared using BLASTX [5] against a database of all RNA virus protein sequences obtained from GenBank as described previously [2]. Although we focused on genetically characterising three of the viral sequences with highest coverage, our analysis of Trinity assemblies revealed that further uncharacterised virus sequences were present in the RNA-Seq datasets. Primers were designed based on the three viral sequences, and overlapping fragments were amplified by PCR and sequenced by the Sanger method. Apart from the first 202 nt of FfusV-1 and the terminal 59 nt of MsaV-2, sequences of the entire genomes were obtained by Sanger sequencing; 100% nucleotide identity was confirmed between assembled and amplified sequences.

To determine the taxonomy of the three viruses, related sequences were identified within GenBank (5 Dec 2017, taxID = 10239 “viruses”), using the amino acid sequence of the entire ORF encoding the RNA-dependent RNA polymerase (RdRp) as the query in TBLASTN. Reference (RefSeq database) and selected non-reference (nr/nt database) sequences with > 25% coverage match were used for phylogenetic analysis. Amino acid sequences of the RdRp-encoding ORF were aligned using MUSCLE (v3.8.31) [6], and phylogenetic trees were inferred using MRBAYES v3.2.6 [7], with one million generations and default parameters. Phylogenetic trees were visualized with FIGTREE v1.4.3. To determine genome organisations, potential viral ORFs were identified using GETORF from the EMBOSS package [8], and each translated ORF was queried with HHpred [9] against the Pfam [10] and PDB [11] databases and identified based on homology to previously annotated proteins.

The sequence of Formica fusca virus 1 (FfusV-1) comprises 9,851 nt. The genome organization of the virus follows a typical *Mononegavirales* gene order with five ORFs, encoding proteins in the sequence 3′ – N (nucleocapsid, 361 aa) – P (polymerase cofactor, 184 aa) – M (matrix protein, 149 aa) – G (glycoprotein, 527 aa) – L (RdRp, 1877 aa) – 5′ in negative polarity (Fig. 1). The 5′ leader and 3′  trailer of the antigenome are of lengths 104 and 120 nt, respectively. Identification of the proteins encoded by ORFs 2 and 3 was uncertain as HHpred e-values were > 1, but they were predicted to be P and M based on their genome position. Genome coverage was obtained to a mean depth of 28 for the negative strand and 7 for the positive strand, consistent with active virus replication. Phylogenetic analysis indicated that FfusV-1 is most closely related to Orinoco virus and suggested it belongs in the genus *Orinovirus* within the family *Nyamiviridae* (Fig. 2).

**Fig. 1:**
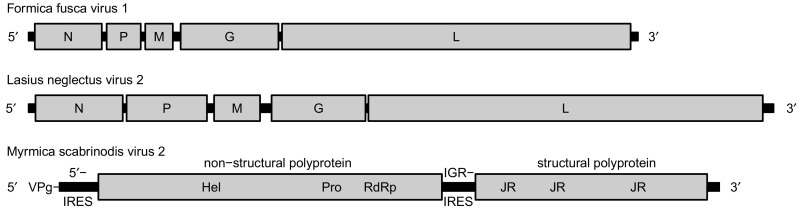
Antigenome map of FfusV-1 (top), antigenome map of LneV-2 (middle) and genome map of MsaV-2 (bottom)

**Fig. 2:**
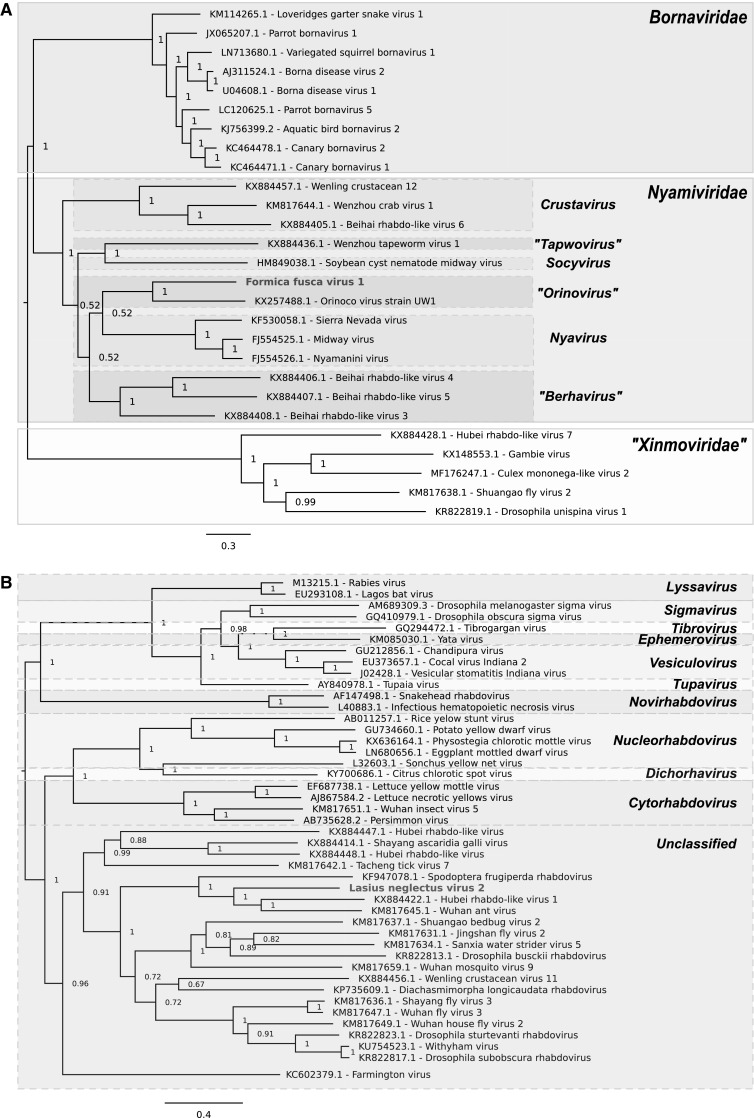

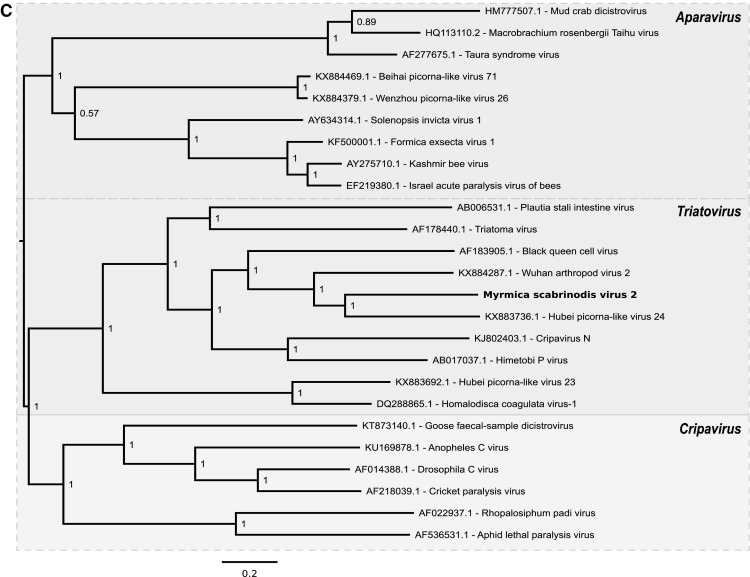
Phylogenetic trees for FfusV-1 (A), LneV-2 (B) and MsaV-2 (C) and related viruses. Numbers at nodes indicate posterior probabilities. Trees are rooted at the midpoint. Leaf labels show virus names and GenBank accession numbers

The sequence of Lasius neglectus virus 2 (LneV-2) comprises 12,041 nt. The 5′ leader and 3′  trailer sequences of the antigenome are of 115 and 174 nt, respectively, and share nine nucleotides of terminal complementarity. LneV-2 follows a typical *Mononegavirales* genome organisation, encoding proteins in the same order as FfusV-1, N (470 aa), P (433 aa), M (248 aa), G (506 aa) and L (2124 aa) (Fig. 1). As with FfusV-1, the proteins encoded by ORFs 2 and 3 were not confidently predicted by HHpred but inferred based on position in the genome. Genome coverage was obtained to a mean depth of 45 for the negative strand and 14 for the positive strand, again consistent with active virus replication. Phylogenetic analysis indicated that LneV-2 belongs in the family *Rhabdoviridae* but does not belong to any of the defined genera within the family; instead it falls within an unclassified cluster of arthropod-infecting rhabdoviruses. Based on the phylogenetic tree, LneV-2 is most closely related to Hubei rhabdo-like virus 1, Spodoptera frugiperda rhabdovirus and Wuhan ant virus (Fig. 2).

The sequence of Myrmica scabrinodis virus 2 (MsaV-2) comprises 10,663 nt. It contains two ORFs of 5553 nt and 4089 nt in length separated by an intergenic sequence of 196 nt and flanked by 5′ and 3′  UTRs of 630 and 199 nt plus the poly(A) tail, respectively (Fig. 1). HHpred analysis indicated that ORF 1 encodes the non-structural proteins helicase, protease and RdRp, whereas ORF 2 encodes three jelly-roll structural capsid proteins. Genome coverage was obtained to a mean depth of 262 for the positive strand and 0.2 for the negative strand. Importantly, the negative-strand reads – although sparse – were distributed throughout the genome, indicating the probable presence of a full-length negative strand. Phylogenetic analysis indicated that this virus belongs in the genus *Triatovirus* within the family *Dicistroviridae* of the order *Picornavirales* (Fig. 2). In dicistroviruses, the second ORF starts at a non-AUG initiation site due to the presence of an unusual intergenic region internal ribosomal entry site (IGR-IRES), which directs translation of the 3′ ORF via an initiator-Met-tRNA independent mechanism [12]; a typical IGR-IRES structure was predicted in MsaV-2 and used to infer the non-AUG start site of ORF 2.

## Electronic supplementary material

Below is the link to the electronic supplementary material.

Supplementary material 1 (DOCX 29 kb)
