# Supplementary material for: Discovery of three RNA viruses using ant transcriptomic datasets
Source: Arch Virol. 2018 Nov 10;164(2):643–7. doi: 10.1007/s00705-018-4093-2 (PMC6373249; doi:10.1007/s00705-018-4093-2)
Supplement: Supplementary file 1 — Supplementary material 1 (DOCX 29 kb) [file 705_2018_4093_MOESM1_ESM.docx]

### **>FfusV-1 (antigenome)**

ACATGCTTGCGTAGCAAAAACAAACAAGCATATTATAGAAAAATGGACCCACAAAACATA

AGAAAGAACCCTTACGTCACTCCTTGTACGAGATCATAAAAACTATGGCACAACAACCCG

CGGCAGAGGTGGCATTCTGGGACACCAGTAAGGATACAGAAGCTTTACCAGAAGTATTTG

CTCTAAAAGTAGCGGAAGCGGTTCCTGCAGCATACATAAAACCAGCACATGCTATACTCA

CAACAGCGCGATTCGAGAAAGGTGAGGTCTATACCACAGCAGTATGTTGGCTACTTGGCA

AAAAACCTAAGATGGTACCCACCAAGGTGTTTGAAGGCACACCTGAGCAAATGAATGCTG

CAGAAAAAGCCAAGGCAAGTCCATCAGCAGATCAGATGCTGGTATTTTACGGCATGACAG

GTCTGGCAACCCTTGTGAAAGAGGTCCGCACTACAGGTACAGCCATTAATACGGACTATC

TGACAAAGAGATGGCAAGCAATATGTGCCACTAACGGCATAACCAATATATTTGCAACAG

GAGCTGAAATGGCTCAGTACCTGGGCGGCATGATGAATGAGGCTGTCAAGTGGCAGGAAT

GGATAAAGCCTCGCGCAGAACTGAGGACTCTACTTCTGAAACTGGCCCTAGGGCCCGGCC

ATGACGATCGCCCAGCGCTAGTGAAAAGTGTGTTGGATCAACTTAGGATGATATTAACTG

ATTTTGGACTTAAATCAACACAGGTAATGTTGGGATTCATCACTTCTGCAAGCAGGGCGA

TAACACTAGCCCCCATAGCACAGCAGGCTGTGGATTTAAAGACAGAGGTGGACAAGTTAA

AGGATAAATACAAAGAAAATTATCCATATATAAGAGTCTTTCCTTTACCTGGTGTTGAAA

AGTTGAATCATCGTTTATATCCTGACCTTTATTATGCAGCTGTTAGTACTGCACTGCACA

ACAAGGAGCTGGGTGTAGAAGGCAGGTACAAAATGACAGATGTACAAACAACCATTTCTA

GAGGATTAATAGATAAGCTAGCCGACAAACCTCTACACATGGAAACAGGTGTGGATGAAA

CCACAGTGGAGAATCTAGAGAAATTGGGTATACATCTACAAAAAAGGAGATATGCTGAAG

AGGAAGAAGAAGACATGCCACCTCTAAGAAGGAGGAGACTTCAACAATAAGGGCACATAC

CAGACATTTAAATCTTGTAAAAAATGACCAACCTACCTCCAACAAAACCCTCAGTCCTGA

CATCATGCAGGAGAACCCACTTGTACCCAGCGATCCAGAGAGTGATGGGGATCTGGCTGA

CACAATAGAACAGCAATACTTACCAAAAACCCCATCCTCTACCTCTACTGCTACAGAGAG

AAGCCTGAGAGCGGAGACCCCTATGAGAACCAGAGCTGCAGGAGTGAAACGCCCCACCAA

CTACCCGATACAGGAGCCGCCACTGACCAAGCGCAAAGGAAATCCCACCAGATCACACAC

TGAGATAATGAATAAGCTGGACCAGTGTGATGCTGACAATAAGACAATACTAGCTAATCA

AGAGGTGTTGCGTATAAATCAAGAAAATATAATAGCACGTCTTGACAGGATGACAAAAAT

GATTATAGATTGTCAAACTAGAATGAGCGTAGCCGAGATTCGCATACAAGGCATGCAGGA

GGCAAGAAAGGAGGCAACAACAGGTCCCCAGGCAACAACATCGCAGGACCGCAGCACGGA

ACCCGATGTCACACCCCACGCACACGTCGGCAGCGGCACAGATGATGCGCCGTGTCTGGG

TGCAGGTGCGTTCTTCTAGTGATGTGCAGGGCCCCCTCTCCTACCCCATCCATACATGCA

CGCACATATCTCTCACTCCTACATTAAATGCTCATGTTTATATTCTTATATTCTTATAAA

AAATCAACTACCAAAATGAAAAATGAACTGATAGTAGAATACAAGGTTCTAGTAGATCAA

ATCACACAAATCAATGCCTGTGACAAAATCCCACAGATAATAAAGGTAGATCTAGAGCAA

GCAACAGGAGCTGCAAAGTGGGAGCTGACGGGACGGAAGGTCTTATTATATGTGAAGGAA

TATTTACAACCTGAAGCCAAGTTATACACCAATATATCCTTTTTGCGAGATTTACAAAAT

CATGGACAGACACCCGCATCAACACTTCTGGTATCAAACAGAACAAGCAACGAGATGCAT

AATTTGTCTGCTACAGGAGATGATTCAGGCATTGCAGACACTTCAGCAGATGCAGATGAA

TGTGCCAGTGCCCTACATAACCTTAACCTCAGAAGAGGAAGGGGAGGAACCCCTTGACGA

GGATGGGGATGATGAATAATCTTGTATCAGAATACTATCATTGAGTAGATTTTTTAGACA

TATATGGTATACACTCTTATAAAAAATTAACCATCCTACCAGACAAGCCCGACCCAAACA

TGAGACCCTCGTGCCTTCTCCTAGCACTGGCAGCTGTGTGTGTGACCGGTCGATCGCCTC

TGTTGTGTCAACGCAAAAGGGGACTACGTGTATTTAAAAAAGATGAACCTCTGAACTGTT

TATCCAATGCTCATGTGCAAACTGTGGAGGTACAACTAAAGGTACCATCCTTCAAAGAGG

CACATGCTTCGGGACACTGGACAGGCCTTTACAAGGCTACCTGTGAGACACATTACTTCT

TCTGGGGCTCTTACACTAGGGAGTTCTGGCATGTGTCAGTGCCTCTGCTATCAACACAGC

GCAATGATATAAAGCAAGGAGGATGCCCCAGCTTGACTCATCCTGTACCACTTCTTCATC

ATCCGGAGCCCTCATGTGTGTACACCTGGCCTAAACAGACTAACACAGAGATTATGTATT

GCATAACACGCCCTACCACCATAAGAAAGTTATATGGCCAACCCTTAACATCAGATGAAG

AACAGTTGGATGAGCGCACAGTGCAGAATAATGGTGCATTTACAGTCACAGGAGCTGTTG

TACATTGGGAACAGAACATAATAGAACCAACACAAAAGGAAGTCAAATACACAGGTGTGG

CATTACTGCAAGAAAATCAACTTATTATTGAGTCGCTGCAGGAAGGCTTCATGCTCCTAC

CTGGACCAACAAATGATGGTCCTTATGAAGTATGGAAAACCATAGAGGGGTATGAGCTGC

GGATACTACAGAAGACAAGAAATAAGAGACAAAGTTATCAAGAAGCCATGCAAGGGGAAA

TTACCTCAAAGTTACAATATGAATCATATATAATGGATCAATACTTACAAAAATTTCAAC

AAACATGCTTTAAGCAACAGTTAACCACTAGAACATTGGATACAATTTATAAAGAAAATG

CGGATGAATATGCACGAGCTCTGCTAAATGAAACAGATATAACCGCTGCAACGGCAGGAG

ATTATCTCTTGGTATGGCCCTGCAAAAGACCTGAAATATGGTACATAAGAGAACAAGAAG

ATGAACAAGTATGTACACAAGATATACCGATCATCTATACAAGTGATGCTAAAAACTACA

CAGGATACTACAAGATAAGAACAGGGCGCATTGTCAATGATACCAACCGCCTGCCCTGCG

AAAGTGTGCAAACCACACCCTTCATGAAGGATGAGCGGCTATACACGTGGGATGGACGCT

GTGTCACGCGCATCTCAACAGAGGGGGTGAAAGGCTTTGCATATCCTAATCAAGGAGCCC

ACATGGCACCTGTATGGTCCAATCACTGGCTATATCACGAAAGTGATTTTCACAGAGAGC

CACCTCTTTATCTAACAAAAAGAGATATAGAATTGCAACAGCTAGTACAGCATACAGATA

CCTACACAAATACAGTGGGTGTTCCCTTCATGAACATGCCCTCCCTTCCCTCTCTGGCGC

GGTGTTTGGAGCTTTTTTCTTTGACAGGAGGTTTTCTTTACTTGATGGGTCTTATCCTCA

CCAGGCTGACGCGACAGAATCGTCCTCCTCCTACGGGTACCAGTCCACTAGAACTATACC

GACTTATGCGACAAAACACATAAATACATATTACATTCTATAAAAAAATCAACCTACATA

CCACACAGACCCCAATCATGGAGGAAAGTCTTTTTGAGACTCTCCAAGAAGAGGGTCTTC

AATATGAGAGAATACATCAACCTATTTTTCGCAAAGACCGGCATCTGTCCTCTGCACTGA

TAGCAACGGAAATCGACTACATGCTCAAGAACAAAGACAATCCTAAAGGGCTGAAAAGGG

AGCACAGAGAACTGCTAGATCACTTGAACAAGACACAGGCATCCATAAAGGACAGTAAGG

ACTTTATACCATGGGTAGCAAAGGAATGGGTCAACATAGCATTCACCAACGAACCTCTTA

CAGGACTCACCACAGCTCTTAAGGTGGCCAGCGAGGCCCTGAGGATCTCTCTACAGGACT

ATGCAGGTATAGGCCACAGATTACATATAAAACCCACCAAGGATCTTGCACTCCTGTATC

ATGCCAAGACTGCTTATAATAAAGTCATAAAGGATATAACATATCAAACTCTATTTCATC

AATCGTATGTCCTGGGTATATACAAGGGAGATGCTTATGGCAACCTTCATTTGATATGGC

AGGATAGCAAGACATGCTGGCTCATAACACATTCCCATTTTGTGGCATCACGTGATATGA

TAAATTCCTGGTTTGATGCACACTTGTATGGATTGGCCGCACGCGGCAAGTATCCCGGTT

ATGACTTTTACAAGGAGATCAGCAGTGTCATCCAAGCCGGGAGGGATTTGTTAATAGCCC

GCGGTGGTGAGGCATATACTCTAATTAAACTATGGCCATCTTTAGTTATATCCTGTATAC

TTAGAGACACAGAAAACAAAATTGACTTTTATCATACCTTGCAAGAGTCACTACTCACGT

ACACAACCTGTACCTTTTATCAATTAGCAACCAGAAGCATCTGTACAGATATTTGTGCAC

ACATGTCTCTTGAGCTTACAGGTCTCTGGAAATGCTTCGGTCATCCAAATGTAAATATGA

GTAAGAGTGTAAAAACATGGGTAAGGAAAGGGTCCTCATTCAAGGGTACCTGCAAGCCCA

TAGCAGACATACTGGTTTGGACTTTTCGCCTAGAATTTTGTAGACAATACTATAGGAGGC

ACAAGAGATGGCCTGTGGTAAGGATGTCCATTTACACACCCTATAAAATAAGACGTGATT

ATTTGAACAATCAGTGGAGTGAGAAGCCATCCACACCGTGGAGGCCCGAAGAATTTGAAC

ATGTTTTCATGGAGAAGAACTTGGATTTCGACTACCATATAGATATGACAGATCTATTGT

CAGATAAATCCATAATACCCAGCCGTGAGCAATGGATACATGAATTTGACAAGCAAGCAC

ATAGAACAATATGGGGTAGGTTTCCCAGTGGACCACCACCCACCTCCAAAAGTGTGGTAG

TGCATTACTTAAAGCAAGATAAAATAACGGTAAAGGAGGTGATAGACAAGCTGCAAACAG

GCAACCTGCCCTTTTCTTGGCGTGTCATGGTGGCTGTGCCGAAGGAGCGTGAATTTAAAG

AAGAGGATGCACGCTTTTACGGTAAAATGTGTTTTGAAATGAGACTCTATCAAACATCCA

CTGAAAAGAATATAGCAGACGGTGTGTTTACATATATAAAACATCAGTCAATGACTATGA

GTGAAGAACAGTTAATACGTACTATTTTACGTATGAACACGCCTGTTATACATTTGGAAG

GCGAAACTTATGTGTTTATAGTCTTAGATTTCTCCTCCTGGTGCACCAACTTTAGGTTTG

AGCTAATTACACCCCTATTTGTAGAATTGGATAATTTATATGGGTTGAATGGTATATTTT

CACTTACACACCTGTTTCCTCTTATCTCAGTATTGCTATTCCAAGACAGGTTTAACCCTC

CTCAACAGCGCAGCAACGGTGATCCCAGTGAGGGAGAAAGATGTTATTACGCCCCCGAGG

CTTGGTTAGAGGGTCAAAGACAGAAAGGGTGGACCCTGGCCACTATTCTTATAATATTAT

TAGCCTCCTGGAAATGTGGTACCTCCGCCTCTCTACTAGGCCAAGGAGACAATCAAGTAA

TCCTCTTAAGAATACCACCTAATGAGCATCTTCTTCAACAAGGCATGAATATGGATACCT

ATGTAAAACATTATCTATCTGTGCTTAAAACATTATGTGAAGAAGCCCAAATAGTTATAA

AATTAGAGGAAACATGGTACTCACGCCATTTATTTGAGTATTCACGTAAATATCATTATA

AGGGTTCACAAGTTAGTAGCTGTTGTAAAAGAATAACACGATTAGCCAGTGAAGCCAATC

AAGTAATACCCTCTCTCAACAGTGACATAGCAGGAATATTCTCCACGGGTGCGGCAGCAG

CGGCAGAGGACTGCACCCCCATGGCAGCCTACTACTGTACGGTGGTGGAAGCGGCCTTGC

ACCTATGGGACACAAACCAATGGATGCGTAAAGAACCCTGGGAATACACCTGTTGTCTAC

TACTCATGACTCGAAGTGTAGGCGGCTACCCAGTTACTATCTATAGTCAGTTTTGCACAC

GTGCTGTTCAAGACACACTTAGCACCAACTTACATCTCATAAAGACAGCACTCAATGATC

CCATCCTGGAAAGACAAATGAGGAGAGTTATCACACTAACACCTGGACAACACAGAGACT

TTTTATCTCTCATAAAAGACCCACAGAGTATACCCCTAGATATACCCTTGCAACCAGAGA

ACTATATCAAACGTGAAATCAAGAAAGGCCTTATGACTTTAATAGTAAACAGAGATGTTA

AGCAACTTTTCACATTAGATACAGAAGCAGCACATGATCAATTGGTGAGGGATTTGTCAT

CTGTGGTGCCCTGTAACCCAAAATTGTTAAACAAGTTATATGCACTATCAAATATAGGGC

TTCAAGAAAAGTGGACTAGTATGTTTGCCAACACACGATCCATACAGCAGGTTGCCTTTA

AGAGTTGGAGTGATGAAGCTGACGTTATAAAAGCTGTACAGAACCTAGAGAAGCAATATG

AAAGATACCTGCAAAATAAGAAAGAGGATTTATTATGTGATGAGTTAAAGAATGCAGCCT

GTATAGGAACATATACACAATTGTTAAGGGAACGAGCATGGAACACACAAATGGAGGGTA

TTACCATGCCACCCCAACAGGAACAAATAAAGGTTAAACGATGGGATCAACTAACGGGAG

ATCAGAGCATGAAGGCTATACTTATCACATCACAGGTGACAGCAGCCCACAAACAAACAA

GTAGAGGCAAGTATACACCCTACTTTGGATCTATTACACAACTACGTGCTAAGAGAGCTA

CATTACAAGTTATAGAGGTAGGGAGTATGGTCTCTAGTATAAAACAATTAATGGAATTAC

ATGGATGGGTAAAAGGCAACAAGCAACTAACAGAATTGATAGAAACCCTAATAAAAGAGA

AGACAAATATAACTATAGATGAGTTGCAGAAGTATACAAAAAAAGTATATTCCGGTGCTA

TAGCACACAGATTACCCTGTCCTGCTCTGAGAAGGGGTGGCATGGCTAATCAGAATTTAA

ACCATTCCAGTTTTTACACCATAACATCTGACACTGCTCTGGAGTATGCGAAAGGTGGTA

TCAACTATGCAATTTGCTTTCAAAGTTGCTTTTTGTATGGTTTAACAGTATTAGCTCACT

ATACAGAGCTAGGCATACCCATATCACGAAGCACAGCACTCCTATTCAGTTGCGTTTGTA

CCTGGCGCATACCACCAGAATCATTTACCCTGCCTTCTGTGACATATAAAGGAGTTGCTA

CAGGTGTCACCATAGATCAGTTACGTCACAAGGATGTGTATGAAAGATATCCGCTTCATA

AACAGGTGGATGAACACATCTCATATGCTGTTATTACTGCAAGAAAATTTGCTGCATGGA

TTGTAAATAGAAGAACCGTTGATAAAATCACATCCCTAGACAACAGAACAATGGAGGAAA

ATCTTACTTTAACCTTTATAAATCTAGCAGAGTTTAGCCGCTTGCAAGTGCCTCTGTTCA

TAAATTCTTTCATTTTTTACTGTATTTTATTTGATCCTCTTTATTTTGGTACCAAGTATG

ACTACTTTAATGAAATATTGGAAGGTGCTGTAAAAAATCCCTATGACCTATTGATAGACA

GTTTCAAGAGGTGTGGTCATATGCAAGCATTGAACAGCCTACATGGGGGACCCACCAAAG

CGTACTATAGTACAGAAGAAGCCAGAACATTGCTCTATAATATAATAACACATCTTTGTG

AGGATTATAGACGTATACTTACCACTGCTTACATCCTTACTCCTGAAGATAAAATGCCTT

CTCTTATAAGAGCTATGCAGGCTTGGTTAACCATAAATGAATTACCCTCTATTCTAAGTG

TTTCCATGACTAAAGAAGACCTAGATGAAACACTTTCTACAACTTATCGTGATTATCCCT

CTATCATACCTGTGTGCACACTTACTGAAGAAGAAACAACAACTTTAATAAGAGCAGCAC

CCATACAGAGGATTCAAGAGCAGGGTCACATGCATGTTGTCTATCATTTAAATGACCCCC

CTACCAGTGTCAACATACATCCTGCACCTGCCTTACCATACTTGTATACCATATTAGAGT

GCCCTTATGCGACTGCTCTAACCGAGTTAAGTGATAGATATGATTTTACGGAGTTGGTTC

GTGACAGAGTCCTTGTCACTATAGGGGACCCTGAAGGATTGTATTACACAATACTAAGTC

ACACATGGATCTACAAGGCAGGGTATCCTTACTGGTCCAACAATCAGCAGTGGGAGAGAG

ATCCCTATGCAATAATGAATGATAAGTGTGCCATAACATGGTCACGTCAACTCTATCTGG

CCAACGGACCACGAATCACCCCGTGCATACCACCCAATAGCCTGGTCCTGCTAACCAGTG

ATATCCCCGTTGAGGCACCACCTGATACACTTTACCTACAATTAATAAGAGGAGGCACCA

CATCCTGGGATGCTCTACATGTAATGCGCTGCCATGCACATAAAGATCCTTTGGAATTAT

GGATAGTACACAGGAAGCAGGGCAAAACCACCTATGAAGAACAATATATAAATATACCTT

TTCTAAAAAGCATACCCCAAACCCTTGATGTCCTGCATGAGGAGCTGAGCGGCGCATTGA

ATAGACCCGCTTGCCTTTCACCGTATGCCTCTGTATCCCATCTTTTGGGTGTGTTGCCTA

GAACCAAGCTGGAGTTGATCCTGTTATTAAAAACACGCATAGCAAGGGCAACCAACCTGT

TGATGGCACGACAAATAAGAGCTACCACCTATGAGGGTGCCATCGAACAGATGAGATATA

AAGCACGCTACAAAGCCACCATAAAAAGAATTGCTACCTATCATTATTTTTACAGAGTGT

TTAAGTGTGCTCCTTGGTTTGCCATTGATAGTCCTACTGTTGTGTCTTATCATTGTCACA

GGCGCAGTGGGGGGGTGTCTCTCTGCTGGAGGGCGTGTGGTGTTGAAGCTGTGTCCATAT

CTATGCATGACCTGCACCTTTTGTACTTTTATACATTTGCACGTTATTGTTGGATTTTGT

TAAAGCTTGATGCTGGTGAGGTGCCGCCCTTGAGGTGTCCGCATATAGTTTTTTCACATA

CCTCAGAATAATTGTCCTGAGCAATGCATCTTTCAAAAAATCACAAATGTAATAACCATA

CCAACAAAAATCTCATACCTTCTTTCCTTCTTTTCTTAGTGTCAATTTTCTGTGTTGTTT

TTTGTATCCGC

### **>LneV-2 (antigenome)**

ACACAAAAAACAATAAAGAAATGACAGAACGCACAATAAGAAAAAACAAGAAGAAAAATA

TAGTACAACCAATATCTTCGAGATTTTTACTCTATTTTTCTTAACCCTCCGTGAAATGGC

TCGCCATATTAATGCAGATGTTGCTGAGGTCAAAGCTGCCATTGCTGAATCACACAAGGT

TCCTCCGAGACCAGGAGTTACTCTTCTGACTGGATGGAGTGACTCAGTTCTAGATGAACG

TGCCAGTAATATCAAAATCCCAAAATGTGTTCAGGCTCTTACCTCACATCCTCACATACT

GTCTCAATTCTTTAACTTTTACTTCAATGGAAAACTTCCTGAAGATCATACTCCCCATCA

GGTGATATATCCTGTTCTATCCAATCTAGGATGCGAGATTGGGGCCCTCCCTTCCTATTT

CTTCCCCCCTGAGATGCTGTCGGAAACAGGATTTTATTCTCCACCACCTTACACAGGGCC

AAAACCAGACTTTCAAGCGCCAATAACCAAAACCAGTGTTCCCTATACACTACCTCCTGG

ATTGAATCCCACCCAAGAAAGATTACTAAAAGATCTGTATTTGGAGACCACATCCCACGA

CCAAGACGTTTTCGTAAAAGCCATAACAAAGCTATATAACGGGTACAACACTTCATTAAT

GACCAAAGAAAACAGAGATTTAATGAACCTAGCCGGATTTTTAGCCTTACTGCTGTTCAG

AGGCATCTCCAAGGATGCGACCCAACTTGCCCGGGGGGTAAACAAGAAGCTCATCAAAGA

GCATATCCACAATTTAGCCGGATGGCTCACTGGAAATGGGTATTCCCCTCCTTGCAAGCT

GTGTATAGATCTCTGTGCCTCAGACTTAGACAAAGGAAACTCTCACTCAGGGAAGATGAT

GACTTTGTTGCTTAAAAGGTGGAAAGACACAGAGGAAGAAGACCGTCAGTCTCGGGGGTT

GATTCCCCAAATACAAGCCACGATCTTAACACATACAGCTGGAAATGGACTTGGATTAGT

AATCTTGATGTATGTGGCAGTCGACATTTTAGGTTTAACTATTGAAAGCTTAGTAGATCA

CACAATGACAGAAATGTCAAAAATGTCGTGGAAATCTCTTGCAGTGTTTGTTCAAACATA

TTTGGACCCAAAGAAACCGGAAAAAACTTACTTATGGGCTAGACTAATTAATGATGGCTA

TTTTGCTGACTTTTCAGCCAGATCACATCCATATTTAGCAGGAATTTTTGCAGGAATTAT

AGACTCCCAACAGATTGTAGGAACTATCAGAGAGGCAGCTTGGTTTAAGCTGAAGCGATT

TCAAGCAGAACAGGGCTACAACTTAGGAAAAGCAGTGGTTGCAAAGATGTCCAAGTTTGT

AGACAGGCCTTTAACTCAAGAGGGACAGGATGTATTCACTTATTTGCAGATACCCATGGA

ACCCTTGAGCTCTGTGTCTCCCCCGGAGACAGGAGAAGATCCGTTTGCTGGTGTTTCTAG

GATGGAAACAAATCATGAGTCACTGTAAATGTTCAACGATATAGAAAAATCAAGAAGATA

AATATTACCCTCTCACCTGCGACTGCATGTGGAAAAGACAGCTACACGACATATCTATGA

CCAAATACTCCAGAATGTCTTCCGGCAAATCCTCAAGCTTAACTCAAAAAACCTCGGAGA

CAAAACCCAAAAGACCCTCAACCCCCTCAGGTCATAAAACCCCCAAGTCAGAATCCTCAT

TATCCTCTATAGATGCTTTGGAGAAAGAACAAGTCAAGAAACTTGTCAAACACTCCAGCC

TTGATTACATCCGGCAATCCCTATCCGGCTCAGCTGATATCATAGCTGAAACACTAATGG

AAGGGGATGACAATGGGGAATCAGGAGCATTCGGTATCACATTGCCCGCCCCGCCATCTA

CTGCCCCGACTCGAGACCCGGAACAAATTGCTCCCGTCCAAGAGATAGATACACTAACCT

GGTATCCCTATTATCGCACTGAAACTCCTAAAGGTCACAACACTCGCCTATATCTAGAGA

TGCTCACTCGAACTCGGGAAGCTGAGTTAGGTAAGCTCGAGCTGTTGCGATCTACAGAGG

AAGTAGGGAAAACAATAGGGTGTTTAAAAACCCTTATGCTATGGGAACAATTAAATTCCA

TATTTAGATACATGGAAGGCTCTGAATTTCCTGATATTATTAGCGATCTCTGTTATTATA

TGACCACCTGTCCGGACTCGATTGAACCCTATTTAAAATGTCTTAACAGGGTCAAACCCT

ACCTACACAATCCTGGGGCCTCCATAAAGCTTATGGAAGAAAAAGCATCTGCGATGGCAA

ACATCTTTTCTGCACTAATTGACGAAGGGAAAGCTCATCAAGATGAGTCCATCAAGTTTG

CGGAGCAGGTATCAAACGTGTCAGCCAATCTGGCAGGTCTTCAAGAAGTGTTAGAGCAAA

CCGTGATAAAAGTTTCAAAATTGGACTTACTACCCGGGGATCTATCACCCCCAGCTTCTG

GAGTCTCTATTCCCAGCACTTCTAGGGCTCCCGAAGGAACCAAGGTGAAAGAAGTTTCAT

TTAAGGAGCCAGGGGAGTATCGAGGTCACTATCACTTCAAGGTGCAAGACGGCAAGATCA

AATCGATCATACCACCACGCGGAAACCCTGAGATTGAAAAGATGCTCACTTATCATCCTG

ATGTTCAGAAGATTTTTGTAAATCTCGATCAGCAGACACTTATCAACAAGCTGAACCATG

ACCCTTCCCATAAGACTGCATTCACTGCTCCGCAAAGTATGCAAAGGTCTGAACTCCTTA

GAGCTTTGTGCAGAGGTGTTCCTAAGACTTCATATCAGTGGATCAAATCGACATCTCCAG

AAGCCTGATGCTACTGAACAAGTAAGCCCTACCACTGACTGGACCTCTATAAAAAGACTG

AAACAATGTGTTCCTATTATGTTCATAAGATTTAGAAAAACCAAGAAGGGAAATAAATAA

TGGAGGTCATCAAGAACATGTTGAGGCGAAATGCCCCTCACCATAAGATGGAGCCTCCGA

ATAAAACATTGCCCTTAACACCGCTAAGTCCGTTCAAAACTAGAATATCAGGATCAGTTA

GAATCGAAGGACCAGTTAAACAAGAGTGGATAAAGGACATCTTAAAAAGGATTGCCCTGA

TAATTGCCTGTCAAATAGGATCTTCCGATCCTAATTGGGAACTGACCACTCCTTATCTCT

TTGTGGCATTCAGAGCATTGTTCTCTAGAAATTTAGTATACAACTCTTTAAAGGATGCCA

ACGTAACTTCAAGAATTGCGACTCAAATTGACGACAAATTCCTCTCTTTGTTATCTCACA

TTCCAACCTCTTCCAAACCTCATATGGAAATTGTGTCCCATAAATGGGAGTTTGAAACCC

CAGCAAAACGGAAGATCCTGTTTGCTTATTCCATCGAGTGCACATTGGAACCATATGATT

ACACGCTTTATGACCAAGTATTATATTCTGGATTCAAACCTTTCCCCGACATAGACTTAG

AACATGAGTCCATCTGTGATTACATGTTCATGAAATGCTCGGAGCGCCCAATAGATCCAG

ATCATGCTCTCATGGTTCTAACCCCTCAGGTCAGTAGATTGAAAAAATTACTTCTCTCAC

ATACACGGCCTGTTAGTGTATTTAAACATAACCTTACTCATTCTCATTTTCCGTCAGCTC

CGCCATGCGAATCAAATCTCCCCTGAGTCCATGACCGAAATCACCTTATCACAGCCAGCC

CTATCGTTGTTTTCGAGAGTGTTTTATAGTTGCCCAGCAAGTTTTATTTTACACAAGTGT

TTTATAAATCGTTTTTTCCGATCCTCAATATCATGTTGTGAAAACCAATGATTTAGAAAA

ACCAAGAATCAAATACTCCCAATCCACAATGCTAAACACCCTCTTAAAGACACTCCTGCT

TGGACTTGTTTCAGGAATGAGGCTTTCAGTTTATAACATGAGCACCCCCTTTCTTATTCC

TCTCCCCTCTCCACCGGATTGTCACAGCAACAAAAACCATACCAACATTTCCGCAGAGGC

AGTAACCATATGGAACATAGACACCTCTCAATTAGCCGGGGAAGCGATTGTTTTATGGAC

AGAGCAATACGAGACGAGGTGTTCCACCAATTTTTGGGGCAACTATTTGGAGGAGATAAC

ATCAACTGAACTAATTCCCTCTGCTGTCCCTACTGTCCAAAATATGAAATTATGGAGCTC

TCGCCTTCAATTATACAATACACGTTCCACCACAGCTGAATCCATATATTCGTGTAAATG

GATGTCGACCATAACCAAAACCATTACGAAGACTTGGGTGAAAAAATCATCCCTGTATTA

CAATTCCGATGGTTCCCTATTTTCAGAAGGAATAGAGTGGAAAACTAAAATATCAGATAA

CATCTATCAGACTGGATTGAGATTTCTATACTCATCAACCTCAACACTTCATGCGTGTCC

GCCATTGTTAAAACAAACTTATCCTGGTGTAGTACAAAATTCTGATCAATTCAAGTTTAT

CACAATTCCTCAGTTACAAATTCAATTCAATCTTATCATCTCATCTTCACATGCTGTGTG

CAAGGTCAACAACACCACGCTTTATCAAACTCCAGAAAAATATCTGGTATCATTGAGCTC

AAGGACAAAAGATGAGGGGATCTCTCCTATGGTTTCAGTCAACACAGACTACTCATGGTC

TCATATACTGTTTGATTTAAATGCCTTAGAAAAAATGATAGAATCAGAATTGGAGCTCGT

AGAGCACGAGACATGTCTGACTCGTCAGATGTTATGGAAACAAATGTTCTACGCAAGGAA

TCCCTCACTAATTGCACAGTATCACTCGGGAAATCCATGGGCTACAGGCTCATTTATCAA

TGGGAAAATTGCAATTCAAATCCCAAAATTAGTAAATTCCCACTGTACATATCCCAAAAT

GAGACATGTCAATGGCACTCATATGAGGGTAGAATGTGAAAAATTAGGAGATGTTTATAT

ATCCCCATGGGACAACTGCTTGTCCTCAAACCCTTGTAATAATATAACAGACTACGGATT

TTGGTTCAAATCATCATCACAACTATATGTAGACTACATCACTGGAGAAGTTCAATCCTC

CACAAGATTCAACTCAATCTCATTTCCTCGATTCACAGAGGTTAATCTCAACAAACTGAC

TAAATATCTACAAAACCTGCCCTTAGAAAACAACTTAATCGCTCTGGACGCGAAAATCTC

CACAACTGAGCACCTCAGTGTGTCATTTCATGATATAAAGCAGTTTGTCGAAAATATTGT

CCATAATATCTGGTCCTCATTTGTAATGTATATCATGATAATAATAATTATCTGTTATGG

TATAAAGCAATGCTTTCACAGTCCTGGTCGCAAGAAGCCATTATATTAATATGATTTAGA

AAAATCAAGAAGACAAATTCAATTTTTGTACCATGTGGGAATTTAATGAGTTTGATCTTC

GACAGGCCTTTGGGGATGAAGAAAGCTTTTCTCCTTTGGCTCCTGCGCCTCCACTGTCCA

CACATTGCAATGTACCATTAAAGTATAACAAACAAAGAGCTGCTATCTCCAAGCCCAGTT

TTGGGCAGTATTCAACCTCTAAAAACCAATACATGAGGGAATTCGAAATCATAAAAACCA

TCTCGGGACCAACAAATGTACAATACTTCGATCTGTGCCATGTGCAGTTTCATCTTTTAA

ACTCCTATACACCAACATCTAATACCTTAACTACAGCTGAATGGGAACAACAAACACGAG

TATCCCAATCTCTATGGAGGGACCAGAGTCTGGCGCTAACCTCTATATGTTCAGAGTCGT

TTTTCCTTCCAGACTATCAGTTGTCAGAAGACCAGAGCATCTTGTTGAAACCTTATTATA

TGAGAAAGGCCTTCTTTGAGGAAGGAGTCCTTAGCTCAGGATCTGATTCTTTCCGCCCTA

CCTTGTGGTGGCGGAAAGAAAAGGGAGTATCTTATTATCGAGACTACAATCTTCTCATAG

TGGCTTCCAAGAATCTGACTTTGATTGTAACCAATAATGTAAACGTATTAGTGTCAAGAG

ACCACTTACTAATACTATCTGACCTTGCAGCTCAACGATACATCTTGCTGAAGTACTCAT

TATTAAGCATGATGCACCCCAAGCCTCCCTTCATTTCACCAGGAATGCTGATTGAATATC

TGGAGTTAGGTGACACTATCTTGCACAAAGGTGGCAACGACGCATACAAGATTGTCTCTA

ATTTCGAACCTTTGTGCCTTTCTTATCTAGTTGGTGATCTTCCTGTTGGGACTAGATCAG

GGAAAACATTTAAGACTCTCATTATCAATGAACTTCATCACAGTTCTAACCTACTGAATG

TGACCCCAGAAGTAGATGCCATAATTTCTCTATTAACAATCGCAGAGACCAACTCCCCAT

CGTATTTGACAGAGTTGTATGGGCTATACAGAATTTGGGGTCATCCTACTATAGAGCCCT

TAGAAGGTGCAACAGCTCTCAAGAAAATAGCGACAAGGGTTCGGGTTACAAATCAAGATC

TGGTTGACAAGATCACCAACAAATTTAAAGAGGAATTTATTTGTAGGTACATAGCTAAAG

AGCATGTGTGGCCAGATCTCAATTTAAAGATGCTACCCTCAGGGAACATCATTGTACAAG

CACATGACAGAAAGTCAGGATTCCCTAATAAGCACAAGGACTACCGAAGGGAAGACCTAA

AAATGGTTCACTTCAACACATTATTCCCGATAGATCCAAAATTTGATTTAATAGAGTTAA

TTGCCGACAAGGCTTTATCACTATTAACCCCTGAACTTGTATCACAAATAATTAAGCACA

ACAACTGCGGGTTGGCAACTGATAGGTCAGTCCTCATTAACTGGTTAAGATCCCCCATTC

ACGATCCAGAAGAGTTTCTCAAATACATTGACCTCCATGGGTTTCCACCATTTGAGATGT

CCAATGGAGTTCGAGAAAAAGAACGAGAAGAAAAGATTAGAGCTAGAATGTTTGGGTTAA

TGACTCTTTTTAAACGGATGTACATAGTTCTAACCGAGGCCTTATTGGCAGAACATATAG

TTCCTTTCTTCCCTGAAATTACCATGATTGATGATGAATTATCTTTGGACAAAAAACGAT

ATCAATTTAATCAAAAACCAGAGAATATGGATCAGATTATCATTAGTCTTGATTTTTCAA

AATGGAATTCTAATATGAGGAAGAATGAGACCCTGCCCCTCTTTAACTGCTTTGATGATA

TGTTTGGGTTCAAAAGTTGTTTTTCTCGGACTCACGAAATGTTTGAAAGTTCAGCAATTT

ACCTGCTAAACGGGACCTATTTGCCAAAAGTAGAATCTCCATTGAGATTGAGACAAGATC

TAGGCTGCTGGTATGGACACTTAGGTGGAATTGAAGGACTAAGACAAAAGGGGTGGACGA

TTTGGACTGTGGGTCTCATTTTGCTAGCTTCTGAAAATCTCAATTTTTCCCTTGCATTAA

TGGGTCAAGGGGACAACCAAATTTTAAGGCTTAGGTTTCCTATTAACACATCATATCGGG

AATCTGAAGATATCATGTATCAATTTTTATCGAATTTGAATAACATTCTGTCAAAAATCG

GGCCTCCTTTGAAATTGGAGGAGACTTGGGCTTCCAGAAACCTATATGTTTATGGGAAGT

ATATCATTTACAACGGTGTAGCTCTCCCTACATCTGGAAAAAGAATTGCGCGCATCTTCC

GTTTATCGAATGAAGACTATCCGACCTTGGAATCAGCTCTGTCTTCTGCCACTGCCAACC

TAACTGCGGCTTTATCATGTCATTATTGCATAGGTCCTCTATTTGTCTTATATTTATCCG

AAGTTGTAGGAACCCTTCAACTTTCCATGAGGTCATGCTATTTGCAAAAACAATCTTTCG

GTGTCACATTTTCTAAACAAAGCACCATTAGAATCCCGGGAGAAAGGGTAGCTTATAGAG

ATGTTCCAAAAATTACAGAGTCTGAATTTTACCAACCAGATCTGTTCTATGAAGCACTGT

GCATTTATCCCCGCGCGTTGGGAGGATTACCGGTGATGACGGTTTTTGATTGCCTTTTAC

GAGGATTTCCCGATGAAGTCAGCTATGCACTCGCCTCTTTGAAAAGAATCTTCCCTTACA

CACATCCCAGTTTACAGAAACTGATCACTCGAATGTGCTCTCCTCCCATTAATTCCCAGA

TGAATTATCAGTTAATTTCTGAGCACCCAACCGTCTTAAACCTGGAAGTTCCCCCAGCTC

CGAGTGAGTCACGGAGAAATTTAATTATAGATTTCGTAAAATCTGGAGACATTCATTTAA

ACTCATATGTGAGGACTTTCTTAGATTTGTTAGATTCAGAGGAGGATAAGCATGTTTTAA

AATATTTAACCACTGTGGAGCCCTTTAATCCCAGACTACTGTCAATGTTTATGGGAGCAA

CAGCTGAATCACGTGCCAGACATGTGGCCGGAAAGTTGCAAAAAACCAAGACCATTGCAA

CTGTTGCACGAACCTTAGGTTCTGTGGATCTTTATGCCAAAATCATTGAGGCTGAAAAAA

ATCATATGGGTTCCATTTTCCGAAATGTATTCTCGGATCACACAAATACCGTAAAATGGA

ATTCATCTGTGTGTTCAGTAGAGCATGCCACAGCACTGAGACGACTGGGATGGGATAGAG

AAATTGTGGGTGTTAGTTGCGTGCCTCCCCATGAGTTCATGTCGTTGGAACTTTCATTGT

TGGACTACACTTGTTTGGAAACATACGAATTGTCTAAGGGGTACGTCTCAATTCGCTTTG

ATCCAAGCTATGAATTGGACAGTTTTCCTTCACCATTAATCAAGGGGCCATTTCCGCCAT

ATAGAGGGTCCGTGACTCGACAGAAAGTGAGCGGATATGGTGACAAGATAGCAGCCCAGG

CTGAACCAATTATTCAGAAAGTTTTAAGGTTGTATTCTTTAGTCGGATGGGGAATTCCAT

CCACAGGCAATTTGCACACCCTTTGTGATAAACTCCTAGCTCGACATACAGATCTTCCAA

GTGAACACTTGTGCCCGCAAGATGAAGAAATAACAGGATCAGTGCATCACCGACTACAGG

ACATGAGAACTGGGCATGGAGGATCAGTTCCAGTACTTCCGAATTATGGCTCGAAGTTAA

TGTTTGACACTTTTCCTCTGGTAGCCTACTCTAAAGGATCCAAAAATGTCAATTTAATGT

TTCAGTCCTTCATGAGCACATCCGTAGTTCTCCTTGGAGAATTGATATCGTTGGGGTGGA

AAAGTAACACACCAGTTATACATTTACACGTGTCTTCTAGCTGCTGTGTTCAAGAGTTGA

GAGAGGAATTATGTGAGTCCCCTGTCCCTTTACCTTTCGAGTTAACTTCTTATCCTGATA

ATCCGTATCTTTATGTCAAGAAAGAAAAGATTTTGATGCTTATGGAGAAAGGATTACGCT

TTCCCATTAATAGGAACCTCGGTCCTGATCCCCTGAGCTTGTTTCACCGATTTCACTCTA

TCTTGGCAGAAGAATGCTTTCATTTATTGTCTCCTCACCACTGGGATCGTCCATCTTTCA

ATTTTCGAACTCAACAGCTCGTAATCAACTGGGTCCTTCCGTGTAATGCCAGCTACCTCC

TTACATCAATTTCCTTGCGACTCTGCACTCACTTTCTAGGAGCTATCAGAGAGAGAGATG

TTACCCGTTTTTTAGCCAGGGTCAGCGAAAGAATTGAACGGGCCCCCATGGAAAATTGGA

AGGCACTTTCCGCCTTAATATTTGCCCCAGACATTCACCATCATGTTGTAGGAAATAGCA

TCTTTAGTAGAGTGTCTGGAAACCCCATTTGCAGCGAGTCTACACTGGGAACTATTCTCA

AGGAATCAGTTGTTTCCGTGATATCAAATTGGGCTCACGAGCCAGACACTAGAAAGAATC

TCAGGTATGTAGACTGCTATGGGAGACCTTATTGCGGGTTAAGTCAACATCCATCCTTGT

TGTTGGTAACCAGAGATTGGATTATGGGAAACTTGCCCTCTGTGGATATACGTGATTGTA

GATATTTGATAATCAACTTCTTAACAACTAAACCAACTGGAAAACAACCATCTGAACTCC

TTGTAGTAGCAGAACATTACTGTAAAATTGGAAAACTCAGAATTTCAGGAGAAAATTTAG

ATTATTTATGCAAAAGAGAAGATATAAAACTAGCAGTCACTTTACCTGACTCGTCCTTAG

TAGACAGTATATCAGATGGCGGTGAAATAATTTTGGAATTTTCCCGTGATCACCTGATTC

GAACATCTCATGTAGTACAGAAAGAACTTATCTTCTCCAAGTCTACGTCTTACGAAAACC

ATTTAATGAAACTAGCTTCCACTCCAACAACAGGAACTTACAAATGCCTCTCTTTCTTGA

ATCTCATTAAGATTATCAACCCTCAATTTATCGGATGCTTAGGTGATGGAGGAGGAGGTT

TTACATTAGCAACCTTGCTTTATTACAAAAATGCAAAGGTTTTTTACAACACTCTGATAA

CCAAAGAAGTCCCGATACAACAGAGTTCACCAATACCCTTTATTCCTTCACTGGCAGGAC

ACAAAGATCTTGAAAACAGGATAGTGGGATTAAATATCACCACAGAAATAGTATCCGACT

TGACTCATAAGGATCTAGGTATGCATTTGGAAAACAAAGTTTCCGCCAAATTTGATTTAA

TCTTGTGTGATGCTGAGTTTACAAAAGAGGACGCAATTGAAAAGGGTGTCAATTTGGTGC

GGGGGGCCACCAGAGTAGCACGTGTTGTCAATTCTCCCTACTTAGTTTTCAAAACCTATT

TGAAAAACATCTCTTTAATTTCCTTTCAAATTAGTTATTTACTTTCACATTATAACTCAG

TTAAAGTTTTTAGGTCATTTTTCTCTTCAACACATAATACAGAAATTTATTTAATTGCCA

ATGACCTTGGCCCTGAATTGAGTTTAAGTTGCATTGAAACAAATAACTGGAATGGTTATT

TCTTGAAGCCTCCGTGTGTTGATCAATTATATCAATGGAGAGATTATTTGGTTCAGTCTA

TAGGGGTAGATATAACTGAATTGTCAGAGGAGTATACTAAAATTATTCAACCTGAAGATA

ATCTAACATTTGTGACTGAACTTAGAAAATACCTTCCTTTTTCTAGGGCGGGTCGCTCTT

TCATTTATCCATCAGACCCTGTGAAGTGGATTCATGACACATCATCAGAGTGGACAGGCA

AGCCACAAATAACTAGAATTCGCCTTGAGACTACTACACTTCATCACTCTTACTTACGAA

GGTGGGCTATTTGTCTATTACTCTCGTATTTGATGACAAACTTAGAAAACCTACTGCCAA

CACTTCCAACTTTGCTCGAAACTGGATCCTTAGTTTGGTTTCGATTAAGAAATAAGTCTT

GGGACATTTCTTTATATTTTGATCCAGTGGATCATATTCCCACTGCCTCAGGAATTAAAG

TGTATTCTTTTGAGCGGTTATTAACTACCTCAGATCTTAAATTGATCTATCGCCTTCTGG

GTATTATGATTTATTTGAATGTCAGCTTTCGAAACTCCAACATATCAAGTCCCTTCTACG

TGCGAGGAGACTTGACTGTTAATCACAAACCTCTTAGGGATCTTCAAACAGCCGAAATTT

CCTGGGTTCGCGACAAGATTCAATTTGGAACAACCAGTAAGGTTCCCACCCCATCCAAAA

AGCAGGCAAAACCCCTCCCCATACAACGAAGACGTCACAGGCTCTAAAACCCTCAACAAC

TCCCCTCCCGAAACATCAGATTTGAAAAAATCAATTGAATTGCATAGGGGAAATCAACTT

CTAAAAAACAATCCCAACGATCCTGCCACAAACCTTTGTCCTCGAGATGTTGTCTATTCT

TGGAGTGTTATTGCGTGCTTTTGTGATTTTTGTTTTTGTGT

### **>MsaV-2 (genome)**

GGGAATCCCTATGCGCAATTATTTGCAACTCCGACGTGGCTGAGTATAAACGATCCCGTA

TGTCCTGAACATGACAGCTTAGTCAATAAACCGTTCACCCCAAACTCATCAAATTTACAG

TATTTAGATGTAACTAAAATCTTTCACTGGATTAACCCAATTAAGAAAGATGATTAGTAT

AAGTTAGTTTTAATTATTAGTGGAAATCTATTTCTTTTCCCGTGTATAAGAGAGACCTAT

TTGCACGTTAAATATTTAAATGGAATACATGATGGTGTCATGTTAAAAACACTCTTACTA

CTGGCGTAAGTATTCTCGCTTTTAGTATCGTATTAAAGAAATACCGTATAAAAGATTGTG

TATCGCGATTGCGAAAGCTAAGACAATGGGTGTAACCCAGGTCGATAACCATTAGCACGC

GGCATTTGCAGCAAGCAAAGCACTTCCCTTTTATATTGCCTCATACTCGCGAGGTTCGAC

TATGAGTGTATGGAGCATGAAATTGAATTATCTTTCATGTTCTGTAACCTTCGCTTGGGA

AGGACGGATCCTTTCTATTTTTATTAATGACAACTAAATCTTTTGTTCAGTTGTTATCAA

AGCTATCAAAGTTTAAACAAATAAATTAAAATGTCTTACTCAAAGAATATTTCTCAGCCG

TGGGTTAAGCAAAATTTGGCCAAAGAATTTTTGGAAAATGTTGAATTTCAACGTAAACAT

CCCTTTCTTGCGGAATGGAAGGAGTGGCGACAAACAGTGGCTAATTATTATGATAAACAT

TCTACTTACTTTGCTTCTTCTATTACACGTGGTCTTCCAGCTAATTGGTATTATTATACT

GGTAATCATGAAATTAATTGGAGTGAACTTTCCTTTTCTATTCAATATACTTCAAAATAT

TGGAAGGTTGGTTCTCCACGTGCTAAATTTTACGAAATTATTCATAATATGCGTAATGAT

TGGGATTCTGGCATTTATGACGAAGACTGTCAATGTCACAATAGTCTCTTCGATAACGGA

AACAATGAAATTATCTTCCCGTATGAAGAATGTAAGTGTCAACGATTCCATTTACTTAAA

CTTTATTGTTCGCTTATATCTCACTATTTTAGAAAGTCTAGGAGGAGTTATCGTAAATAT

TATGTTAGATTAATTAAGATATTATTTTCTCTTCCCTTAGATAAGCTTTTAAATTTAGAT

TATGAAATGATATTTAGTCCACATAAATTTTCTTCACATGGGCGTAATTTGTTACAGCAA

ATATATGCCCGTGGTTATTTTGATAAATATACAAATGACGATTTTAATAAAATGCCTTTT

GTATTTGAAGAATGGAAACATAAATATTATAATCCTAAACAACAGAGTGGTACGGATCAG

TGCGTAAGTGCCTCTATGTTTGATAATATAGCTTATTTAGCTTCAAAAATTCCAGAGCAG

CAAGGATTATTTCCAGCTAATATTAATATGGTTCATGATTTTGTTGCAGACAGTCCTGCA

TATTCAAAGTTTGAAAAAGCAGTAAATGATACACAAGAAAAATTCTTTTCGGGTCTTAAA

AATACTTTATATTCTTCAGCAAAAGAAATAATGATACTATTTGTTTTTACTGCTACAATT

GCAATGCTTGGATATACCGTAGTAAAATATGGTCAAAAAGTAATACTTAAAGCTCTAAAT

ATGTTATATAATATTACATGTGGTACATTTACTGAGCAAGATTTATCTATTAAACAACAA

TCTGATGGATTAAGCATTCCTTTTATTCCTGCTATGATATTAAATAATGTTATTTCTCCG

CCTACCCACATTTTATCTAAAATTTGGAATAATCCTCAAACTGATAAAATAATGCGTCGC

ATTGGATATCTTGGTGATCCTAAGATGTCTAAGGGAGTTGATAAAATTTCAGATTGGATT

AAACAAACTATAAATAGTACTGTTAGATGGTTTAAGGAGACAATTTTAGGTATTGCTTGT

GAGGAAGATATTGAAAGTGAGTGTTCACCAGTCCAAAAATGGCAAGAAGATTGTGATCAA

TTCTTTAATATGTATTTTGAAGGAAAAATGCAATGGAATGATATGAATTGGTCAATTCTT

ATGAATTTATATGGACGTGGTGTTGCTTTAACTAGACAAACTGCTTTTAATGAATTTAAG

CAAGACGTATGGAAGGTTGTTTTTAAACTTGGAAATATTTTGGAAAAGTTTAATGCCCAC

GGGAGAGTAGGATCTTCGGTTAGGAATCCTCCAGTTACCATTTATCTTTCAGGAGGTACA

GGAGTTGGAAAATCATCTATTACATACCCCTTAGCTGCAGAAATTTTGAAAGGAATATTT

GCTCGTGAACAATCTCCTTTAGATCTTAAGAAATATTGGAAAAATTTGATTTATATGCGA

TCTTCTGAACAAGAATTTTGGGATGGTTACGAGAACCAGTTAGTAACTGTATTTGATGAT

TTTTCTCAGTTGGTTGATAGTCAATCTTCTCCAAATATAGAATTGTTTGAAGTAATCCGA

GCAGCTAATAGTTTTCCCTATCCACTTCATATGGCTTCCATTGATCAAAAGGCTACAACA

ACTTTTAATTCAAAAATTATTTTGGTATCCTCGAATTTAGATAAGCCCAAAACTGCATCT

TTGAATTTCGAAACCGCGCTTTTTAGAAGGTTTGACATTTGTGTTAAAGTTTCGCGAAAG

CCTGGAGTGAAAACAATACCTGGAGTTTTTGATCCCACAATTTATATGTTCCAAAAGTAT

GATATGGTAACTGGTGCCCTGGGAGAATTTGTTTCTTATAAGGACATTATTTTACAATCT

GTAACAGAATATTATAATCGTAAGGGATTCGTGGATACTATGGATGATTACATTACGAAA

GTTTTGAGTGAGCCTGATGAAGTTCCTGTAGAACAAGGTTTAGGAACTGCGTTAGGTAAT

ACAGCTTGTGCAGTTAAATTAGGAATCAAATATGGTATAAATTCTGTTCATTCTAATTAT

ATTGATTTTAAATCTGCTCTAACTGGAGATATCCATCATAAATATTGGCTGGAAGCACGT

TTGGCTCTCGAAAATTTAAAACTTAAGACGATTAGAATTAAATGTATGTGGGCTCAATTC

CGTGAAGAACATCCTTATTTGGTCAAGGCAATGAAGTTTGTGGGCATTTTAGCCTTAGTA

GTTGGTGTGCTTAAGCTTTATTCGTCCTTTACTAAGTCAGAAAAGAAAGAAAAGTTTATG

AGTCCTGAACAATTTGTTAAGGGATCTACAGAAGAGAGTTACAATCCGCCTCAAGTGAAA

GGTGCTAAAGTCGAATATCAAGAATTGTGTAATTGCGAATATGAAAAATCTGAAAGTAAA

ATAATAACTATTATTAAAAATCTTAAATGCTCAGTTTGCAAATTATTACATATCAACGAA

AATCGAGTTATGCCTCCTAAAGAACAAGGATGGTATCAGAAAATGAATTTAAAGATTCAA

GATTGGATGCATGGTACGAAGGAGGATAGAATTAATATGGCTGAAGCAGAAAATTGGGTT

CATGAACAATACAGAGCATCTTGGAAGCTTGCTGGATCACCTAAATTTTATCGTATTGTT

GATGAGGAAGTAGTTCCCGTACAACAAGGATATAATCCACCTCAGGTAAAAGGAGTTAAA

GCTGAAGGACCATGGGGTTTAGGTTGGTGGTTTAAAGATGTTTGTATACATGATCTGAGT

AAAATGTCGTATAGTGAACTTTTTGTGGGACGCAATAGGCATTGGCTTGTAGATTATAAC

CAGAATTGTGAACAATGTGTTGTTCCTCAGGTGTGTCAAGAGCAAGGAGTTAAAGATATA

AATGCTTCTGAGATGATGATGAAAATTATTCGTTCTAATTATTATAAAATATATCATTTG

GACAGTCATGAAGCAATTGGTCATGCTTTGTTTTTGAGAGGGAAAATTGTTATGTGTCCT

AAGCATTATATGTCAGCTTTTAAGAAAATACAAGGTATGGGAGGATCAAATCGAATTTAT

TTCAGAAATGTTTTCCTTGATCGTGCTTTCGAATTGGATGTGAGTGAAATAATAACTAAA

GCGTTTTCTCTAGAGTCTCCAGAAGAACAAGGAGTTCCTATCTTGTCTCGTGACATTATG

GCTTTCCCTGTGGTAACAGCAACGTATCATAGTGATATTGAACCTTTCTTTGCGGAGAAG

GATTTATTGTCATATACGAAGTCAACGGATGTTATGTTACCTGTTCTTTTGAACAATAAT

GTTACTAAGAGTGAACGGAGTGTTGTCTTGTTTCGTTATGCAAAGGGACATTCTGCTTTA

GGAGTTAAGCCATCCACAAGTATTGAGAATGATGAAGGAATTGTTGTTCGCATTATGCGC

AATTTGTGGGAGTATTCTATGGATACTCAGCCAACGGAGTGTGGTGCTCCACTAATTGTG

CGCAATGTCAACATAGCCCCTGGGAAAATAGTAGGCATGCATGTTGCTGGTGTTGAGGGA

TCCGGTTTGGGTTATTCCACTCCAGTTTATAAACAAGATATTCGTAAAATTTTGGAAAAT

TTTTCAAAATATGATACAGTGGAGTTTCGGCTCAAAACTAAATTGAATCCTTATCCTAAA

GAACAATGTCAAGTTCCCGAAGAAGCTGAGTTCATTAGATTGGGAAGTGTGGATAAATCC

GTTGCCCAACCTGTTAGATCTAAAATCTTGCCTAGTCCTATTCATGGCGAAATTCGTAAA

CCAATTACCAAACCATGTGCATTGCGACCGGTTGAGGTTGATGGTCAAACATTTGATCCC

CGGAAATATCGTTTAGGAAGACTTGGAAATATACCTCAATTTATTCGCCAACTTGAAATT

GACTTTGCACAAGAAGCACTTGTAGACGAAATTTCTGATAAGATTAAGAATATGGATTTT

GGACCTAATATTAAATCCGTATATACCTTTGAAGAAGCAGTAGCGGGTATAGACGGTGAA

GAATATATTAATTCCATAAAACGAAATACTTCACCTGGTTATCCTTTCGTCCATATGAAG

GGTTTTGAAACCCGTAAACAAATTTTTGGTGATGAAGAAAGATGTAATATTAATGTGCAT

CAATGTCAAATTATTAAGAGACGTGTGGAAAATATTATAGAAGAAATAAAACAAGGAAGA

GTTTCTGAACATATTTTTATGGATACATTGAAAGATGAGAGGAAACCTATACATAAGGCT

CATAAAACTAGATTATTTTCTGCAGGACCTTTAGATTATTTAATTGTTTGTAAAATGTAT

TTTAATGGTATCGTAGCAGTTCTGCAAAAAGCTCGAAATTTTTCTCATGTCTCAGTAGGA

ACAAATGCTGCTTCATTTGATTGGTCAAATATCGCCAGGGAATTGTTGCTCAAAGCTGAT

AATATAATTGCTGGTGATTTTGAAGGATTTGATGCGAGTCAAGTCGTTCAATTGTTACAG

GCAGCTGGTATGGTTTTGATCAATTTGTCTAAACGATTTTGTGGAACGACAGATGAGGAG

GCATATATTATGTGGTGTTTGTTAATTTCTTTATTTAATTCAACTCATATCACCGGAAAT

GAAGTATATATGTGGACTCATTCGTTACCTTCTGGCCATTATTTGACAGCAGTTATTAAT

TCTATATTTGTTTTGTTATGTTTCTGTATCGTTTGGCAATTGGCATTGGAAAAAGCAAAT

TATATGACAGCACGATCTTTCTTTAAAAAATGTGGTATCGTAGCTTATGGTGATGATCAT

TTGGTTTCTATACCGCAGGAATTTCTTCCTATATTTAATCAACAAACCTTAATATCTTTA

TTTAAACGAATTGGATTGGGATATACTATGGAAGATAAAGATGCCGTAGCTGATGCTCCA

GCTCGACATATTTCTGAAGTAGCGTATCTGAAACGCAAATTTTTATTCGATAATGATCGC

CAATTCTGGCTTGGACCTATATCTATGGATACAATTTTGGAAACTCCTATGTGGATACAT

AAGTGTCCTGACCCTAATGCTCAAACTATTGAAGAATTAGACAGTTGCTTAAGGGAGCTG

TCTTTGCATGATTGTGATACTTGGAATAAATGGGCACCTGTACTAATCTCTGCTGGACGA

CGACTCGGCCACTATACTGAGTTTGTGAACCAGGAAGAGACTAGGGCATTTGTCCTTGAA

TAGGCCCTTATGATGTGATCTTGCACGCGACGGGTTAAAATGTCAGTTTGTTAACGACGC

GTGTAGTGCTATTATAAGAAGAGCGTTAGATATTTATCTTTACCACTCATGATGCGCTGG

TGCAGCCCACCTATATCAAGAGAACCTGACTAGGGATTTTTGCATTCGGTTGTGCTTATC

CCGAAACTACCAACGACCGGCTTCAATTAAAAATACTTCAAATACACAACCAGAGCATGG

AAATGCTGAAGATGCACCACAGGTAACACAGACTGTGGAGGAGCAAGTCCAAATTGTAAC

ATTTGATTCCGATTTGGCAGAGGTACAGGAAGATCTACCTTTCCCTTCTCAAATACCTCT

AACACCTTCAATTCAACATACTGATGATTCTGTTCATTCTGTAATTTCTTTTTTAAAGAG

ACCGCAGCTTTTATCTTCTTTTCGCTGGGCGTCTACTGCAGAGAGATCGGCGAATCTATT

TACCCCAGCAGGTCGAACCGATGGATTATTGGTTCCCACCGGCTTGTTTCAATCACACCC

AATGATCGCCCGCAAACTCGACGGCTTCACATCGTTTAGAGCTACTTGTGTATTGAAACT

TCAAGTAAATTCGCAACCTTTCCAGTGTGGACGTTTATTAATGGCTGCGTCTCCTGTACC

CGGCCTTTTAGGAAATAGAAATAGATTTATATTTTCACATGTTTCAAACGCGCAAAATTT

AAATCATGTTCAGATGGATATCGCTAAAGATACAGAAGTCGAATTGCGTATACCCTTTAT

ATCCCCGTATAATTCTTTTGATCTTATTGATGGCAAATTTGATTGGGCTGAAGTGAGAAT

TTTAGTCTATAGTCCTCTTAATGCCGTAGCTGCAACTTGTTTACAATGTTTAGTTTATGG

TCATTTTGAGGATATTGAGATGGGAGCTCCCACTTCAGGTTCAGTAAAACAACAATCAAG

GATGCCCTCAGCACGTGCTATAGATGAGACCAAGAAGAAGGAGGCGAGAGGTGGATTTAC

CGGAGCATTGGCGCAATTGGGTTCGTCTGTTATTAATGGCGGTGCGAAATTAGCAATTGC

GGCAATGGGATGGTCTAAGCCTATTCTTGCTTCTCCTTCTCAAGTAGTTTTGGAAAGACC

TACTGAAGGTTTCAATTATATGGATGGAGTAGATCAATCCATAGTCTTAGGTATGACAGG

CAGTAATGCAATTGAACCAATCCCTGGATTAGTTGGTACTGCAATAGATGAAACAGCATT

TAATACTTTGAAACGCATTCCTCAAATGGTTTCCGCTTTTACTTATAGTGATAAAATAAT

AACTTGTGAAAATACTGATCGTAATCCAGTTATGTTGTGGCAATGTGCAGTTTCTCCAAG

TTGCAATGTTCCTGCTTGTTATTATTTAATTCCTCAGGCTGATTCGACGACTGGTGTAGT

TAATCCTTATCAATTATCTTGGAAGCAACCGACTACATTAAATTATATTACTGCACCATT

TCTTTACTGGACTGGCTCGCTTGTATATACTTTTAAATTTATTAAAACAGATTTTCATTC

AGGTCGTGTTGAAATTTCTTACCATCCTTTTGTTAATACCGTTGCCACTGATAGGTTTGA

TTATGTTTATCGTACTGTCGTGGATTTACGTAAAAATTCGGAAGTTTCTGTTACCATCCC

ATATATCGCCGCGCAACCTTGGAAGCGTATATCAACATGGCTAGATCCTATAAACCCAGA

TCCTCCCGCACCAGGACGTTTGAAGGATGTTATTTGTGGCATGCTTTATGTTAGAGCTTT

AACACCCCTGATATGTGCAACCTCCATTATATCACCCAATATAGAAGTATGCGTAGAAAT

GAGAGCAGGAGATGATTTTGAGGTTTCAGGACCTGTTACATCTAAATTCTTACCTTTTTC

TTTCCAAAATCCTAAACAACAAAGTGCAGATTTTCGTGTCCCATCATCAATAGGGCGCAT

ATATGAATATTGGGCAAGGGGCACAGCTATTGCTCAAATGCGTGGACTAAAGGATTTTCC

TTTAGTTTCAGTGAATATTAACGGCGATTTAACTCGTGCAAGTAATGAAGCATTTGCAAA

TGCTCGGATATGGTATTTAGTCTTAAAGTGGGAAAATATTTATCCTACTTTAGCTAATGT

AGATCAGGAAAAATATGCTTTTATTTATTCTATGTGGAGTAATGGATATTCTTATACACA

GGATGTAATTATTGACAATCCAAATAACAATGATATTCTTTATTGCTTTCTTCTTCAAAA

TTTTCCTGCTACAAATGTTCCTCCAAATATGTTACATACGGTTGATATTTGTGCTTATGG

TAGAACTCGAAATCAACAAATAGATTTGAATCCCACACAATTTCCAATTCCTACCACAGG

AGGAGGAGGAGGGGGATCGAGTGTTGTTACAATTGATCCCGCGTCTTTACCTCTTAAGGT

AGAAGTTGAACAACCATCTGGTCCACTTAATGTAGTCGTTGAAGAACAACCTATAGAAGT

GAAATTAGACAGTTCACAGGTACCGCTTCCGGTTGCTTCGGAAGGGGGCGTTGCCTCTAC

AGTCAAAATTGACCCATCTCAAATTCCCCTTAAAGTTGAAGTAGATAACGAACCATTAAA

GGCAGTTATTGAGAATACAGTTCGCACAACTATTGACCCTTCACAAATACCTCTTCCAGT

TACTGGAGGTGGCGGTGGTGGCGGAGGTGGAGTTGTAACCATTAATCCCGAACAATTACC

TTTATGGACATCACCAAATAATCTTGCACCTTTAAATCCACGAGAGCAATCGAATAATTC

CTTTCGTGAGAATACAAGGTCCACATCCAGTAAACAGTCCTTACCAGTACAATCATTACA

AGAACGTAGAGGGTTTAAAAGAACCACAATCTGCTTGGATTTTTCCAAGATTGATTCTCG

TGATATTTATAGGCTTTCTCCTGATAATGTTCCTGCTTTTCGTAATGCTCGTCAACAGTC

AGGACAACTGGCTATGGCGGGAGTTACCGAAACGCGTACTAGAGCAATGGAAGGATGGAT

GCCTCCAAGCATTACGGGATCGGAATCAGATGCTCACCGACCCTCCACTGTTAAATGGTG

TACTGGCGAAAAATTCGAAACATATAGACAATATGCCAAACGATTTGCTTTTAATCTTGT

TACTGGTTTGGCTTCTGATAAACCTCTACCTATTCAACCAGTGGAGATGATTCGTCCGGG

AGCATTGACTCTTCGAGTAACACCTGGTACTGTAGCAGGAAATGATAGAACACAGTATGC

TTTGTATGCATTCAATTCTAATGCAGAAGTGAGTGGTTCACCACTAGCTTATGCCTCGTC

AATGTTTGCATTTTATCGTGGAAGTGTTCGTGTTAAGGCTTGGTTGGATCCCCGTGCAGA

TAATGTTGCCATGATTTCTGGTCATTTGGAATATGCTCGTCAAGATATCGATAACAATAT

AACTAATGACACAATAGAAAATTTTATGACACCAATAGCTTATGAGGTTCCTAGTAAAAA

GCAAATTGCAGAGTATCAGATTCCTTATTATTCACCAACTATTATAAGTTCGACTTGGAG

TCATGGCGTAGATAATCAATTTGATGTCCCATTAACAAATTTAGTTATAGGTATACCAGA

CACATATGCTGAAGACACAACAGTAACACCTCCGGTCTTTAAATCTCGACCAACAACATA

CCCTTTTAAATTAGCAGTAGCAGCTGGTGATGATATGGATTTTCATCAATTTATTGGTCC

ACCCCCTGTTATAAATTTAGGAAATTTGACTACAACGGCTAGAATTATTCATTATCCACC

AACAGGTTTTACACCTACTCAACATATTGAACCCGACACAGCAAGGAGTGAAGAGGCAGC

AACAAGATTTGTTCCAGTGGATTATAGCGTGTTGCGTGTTGTTGGAAATATCACGGGAGC

TTCTTGCGGTGATCCACCAACATTTAGAAGAAGAGATTTACCAGAAAATGCCACTGGAAC

AATGGCTGAGGAGAAAGTACTTAGTACACCTCCTAAACAACAACAAACCCAAACATCAAC

ATTAGCGGTAGTAACTAATAAAACTAGTGATAATACACCTAGAATTAGTCAGCACCAGCC

AGTTAGAACTGGTAGAGTCCGAAATCCCCGTGATGTTGAAGGTGAAGGATATGATACAGT

TGATGACAGTCAACCTGTGTCAAATCATTCCCCTAGTAGAGATCACTTATCTTATTATAG

TAATCCTCAAAATTATATTCTCGAGTGAATAAAGAGATCCACTCGAGCGAAGACTAACGA

AGCTTTATTTTGTTAGAAACATTAGTCTGGCGTTTTTCCCAGAAGGTTCAGCACCGTTAT

TTTAACGGTGTGGTCACGTACCCCCTTCTGGGGAGCGTCCTTCTGGTTTTTCCAAATTAA

TTTCTTTGGTTTTCGATTTCTTTATGCAAAAAAAAAAAAAAAA
